# Automated quantitative high-throughput multiplex immunofluorescence pipeline to evaluate OXPHOS defects in formalin-fixed human prostate tissue

**DOI:** 10.1038/s41598-022-10588-z

**Published:** 2022-04-22

**Authors:** Ashwin Sachdeva, Claire A. Hart, Christopher D. Carey, Amy E. Vincent, Laura C. Greaves, Rakesh Heer, Pedro Oliveira, Michael D. Brown, Noel W. Clarke, Doug M. Turnbull

**Affiliations:** 1 Genito Urinary Cancer Research Group, Division of Cancer Sciences, Oglesby Cancer Research Building, University of Manchester, Manchester, M20 4GJ UK; 2 Belfast-Manchester Movember FASTMAN Prostate Cancer Centre of Excellence, Manchester, UK; 3 Department of Surgery, The Christie NHS Foundation Trust, Manchester, M20 4BX UK; 4 Wellcome Centre for Mitochondrial Research, Newcastle University, Newcastle-upon-Tyne, UK; 5 Translational and Clinical Research Institute, Newcastle University, Newcastle-upon-Tyne, UK; 6 NovoPath, Cellular Pathology, Newcastle-upon-Tyne NHS Foundation Trust, Newcastle-upon-Tyne, UK; 7 Department of Pathology, The Christie NHS Foundation Trust, Manchester, M20 4BX UK; 8 Department of Urology, Salford Royal NHS Foundation Trust, Salford, M6 8HD UK

**Keywords:** Fluorescence imaging, Translational research, Biomarkers

## Abstract

Advances in multiplex immunofluorescence (mIF) and digital image analysis has enabled simultaneous assessment of protein defects in electron transport chain components. However, current manual methodology is time consuming and labour intensive. Therefore, we developed an automated high-throughput mIF workflow for quantitative single-cell level assessment of formalin fixed paraffin embedded tissue (FFPE), leveraging tyramide signal amplification on a Ventana Ultra platform coupled with automated multispectral imaging on a Vectra 3 platform. Utilising this protocol, we assessed the mitochondrial oxidative phosphorylation (OXPHOS) protein alterations in a cohort of benign and malignant prostate samples. Mitochondrial OXPHOS plays a critical role in cell metabolism, and OXPHOS perturbation is implicated in carcinogenesis. Marked inter-patient, intra-patient and spatial cellular heterogeneity in OXPHOS protein abundance was observed. We noted frequent Complex IV loss in benign prostate tissue and Complex I loss in age matched prostate cancer tissues. Malignant regions within prostate cancer samples more frequently contained cells with low Complex I & IV and high mitochondrial mass in comparison to benign–adjacent regions. This methodology can now be applied more widely to study the frequency and distribution of OXPHOS alterations in formalin-fixed tissues, and their impact on long-term clinical outcomes.

## Introduction

Mitochondrial oxidative phosphorylation (OXPHOS) plays a critical role in cell metabolism. The mitochondrial inner membrane is the site of the electron transport chain, which comprises of five enzyme complexes (I–V). Defects in the abundance and activity of these OXPHOS complexes have been observed in both normal ageing^1–3^ and pathological conditions, including mitochondrial myopathy^4–6^, Parkinson’s disease^7^ and cancer^8,9^. Accumulation of OXPHOS defects leads to metabolic reprogramming, which may confer metabolic advantages in a subset of tumours for subsequent clonal expansion, and eventual progression to metastasis. Assessment of OXPHOS defects in archived human tissue with sufficient clinical annotation, may help in studying their impact on long-term outcomes.

Until recently, assessment of mitochondrial OXPHOS defects in intact tissue sections was limited to the use of sequential cytochrome c-oxidase and succinate dehydrogenase (COX/SDH) enzyme histochemistry. This method assesses the loss of activity of cytochrome c oxidase (complex IV of the electron transport chain). Rocha et al. (2015) reported this method to be subjective and highlighted the lack of reliable in situ assays for detection of Complex I defects, which forms the key entry point for the electron transport chain^4^. The authors described an improved method based upon quadruple immunofluorescence to quantitatively evaluate frequency and degree of both Complex I and Complex IV defects simultaneously in frozen muscle biopsies. This objective method was validated in a cohort of patients with well characterised mitochondrial disorders and has since been successfully employed to diagnose mitochondrial complex I disorders^5^. Since this assay was primarily designed for the study of muscle fibres, its application in the study of cells with high nuclear-cytoplasmic ratio, such as that observed in epithelial cells, is complicated by autofluorescence and reliance on confocal microscopy^2^. The absence of a nuclear marker also precludes the ability to employ automated cell segmentation algorithms to reliably estimate protein abundance in the cytoplasm. Taken together, these issues contribute to high cost and low throughput.

In order to overcome these challenges, we describe an automated high throughput method employing multispectral immunofluorescence imaging for the assessment of OXPHOS defects affecting complex I and IV in formalin-fixed tissue samples. We demonstrate the use of this method to study the frequency and spatial distribution of OXPHOS defects in benign and malignant human prostate tissue. Additionally, we apply this method to explore spatial and inter-patient heterogeneity in OXPHOS protein abundance at the single cell level.

Multispectral fluorescence allows concurrent detection of up to 8 targets, whilst minimising the risk of signal crossover. We developed a pipeline (outlined in Supplementary Fig. 1) using secondary antibodies with tyramide signal amplification (TSA) that employs catalysed reporter deposition where, in the presence of hydrogen peroxide, oxidation of the fluorophore-labelled or chromogen-labelled tyramide substrate by horse-radish peroxidase leads to deposition of the label. The TSA-fluorophore complex is highly stable and insoluble in water, due to which fluorescence binding and thereby signal intensity, is not influenced by heat-mediated denaturation^10^. Furthermore, TSA is particularly well suited to sequential multiplexing since it (a) offers increased signal to noise ratio as compared to Alexa Fluor fluorophores, significantly improving sensitivity for targets at low abundance^11,12^, (b) often requires lower primary antibody concentration^13^, thereby reducing cost, and (c) helps overcome the requirement for use of combinations of antibodies raised in different species or unique isotypes^10^.

The mitochondrial markers chosen for this study have been employed previously in evaluating mitochondrial dysfunction in various cancers^14–16^ and mitochondrial myopathy^4,5^. NADH:Ubiquinone Oxidoreductase Subunit B8 (NDUFB8) is a nuclear-encoded accessory subunit of Complex I. Reduced abundance of NDUFB8 has been found to be strongly correlated with defects in mitochondrial complex assembly and subsequent loss of function^17^. In line with these reports, NDUFB8 has been used as a marker of assembled Complex I^18,19^. Mitochondrial cytochrome c oxidase subunit 1 (MTCO1) is a mtDNA-encoded core subunit of Complex IV. MTCO1 abundance correlates with COX defects upon COX/SDH enzyme histochemistry, consistent with previous reports demonstrating reduced MTCO1 abundance in COX-deficient muscle fibres^20–22^. Translocase of outer mitochondrial membrane 20 (TOMM20), a well-established marker of mitochondrial mass^23^, was included in the multiplex assay to normalise NDUFB8 and MTCO1 abundance for mitochondrial mass. In addition, we used pan-cytokeratin antibody and DAPI as conventional markers for epithelial cells and nuclei respectively.

## Results

### Optimisation of individual markers using chromogenic IHC and IHC-IF

Primary and secondary antibody concentrations and incubation time were first individually optimised (Table 1) using chromogenic DAB immunohistochemical labelling, then translated to the TSA-based OmniMap HRP system on the Ventana Discovery Ultra platform (Roche Inc). First, adjacent sections were labelled with IgG control antibodies to assess for non-specific binding. Once optimal antibody concentrations were determined using IHC-DAB, automated IHC-IF labelling was performed for individual targets using the Opal 520 fluorophore with OmniMap HRP (Supplementary Fig. 2). Due to low abundance of NDUFB8 antigens, OmniMap HRP amplification was found to be inadequate. Therefore, a secondary antibody conjugated to hapten-based anti-mouse HQ polymer followed by an HRP-conjugated anti-HQ tertiary antibody was employed as an enhanced signal amplification step. This resulted in improved NDUFB8 fluorescence signal intensity (Supplementary Fig. 3). The TSA-based approach enabled use of lower primary antibody concentrations in all markers except NDUFB8, while maintaining optimal fluorescence signal intensity.

**Table 1:** Antibodies used in automated and manual multiplex immunofluorescence.

Type	Antibody	Target	Species	Cat No.	Manufacturer	Dilution
1°	α-*NDUFB8*	Complex I subunit	Mouse IgG1	ab10242	Abcam	1:100^a,m^
1°	α-*MTCO1*	Complex IV subunit	Mouse IgG2a	ab14705	Abcam	1:500^a^ 1:100^m^
1°	α-*TOMM20*	Mitochondrial mass marker	Rabbit IgG	ab186734	Abcam	1:500^a^ 1:100^m^
1°	α-Pan-cytokeratin	Epithelial cell marker	Mouse IgG1	C-2931	Sigma Aldrich	1:10,000^a^ 1:400^m^
2°	Biotin-conjugate	Mouse IgG1	Goat	115-065-205	Jackson IR lab	1:200^m^
2°	AlexaFluor 488	Mouse IgG2a	Goat	A21131	Life Technologies	1:200^m^
2°	AlexaFluor 546	Rabbit IgG	Goat	A175732	Life Technologies	1:200^m^
3°	AlexaFluor 647	Streptavidin	Goat	S1125	Life Technologies	1:200^m^
2°	AlexaFluor 750	Mouse IgG H + L	Goat	A175732	Life Technologies	1:200^m^
2°	Hoechst 33342	DNA	Synthetic	H3570	Life Technologies	1:1400^m^
2°	Spectral DAPI	HRP	Synthetic	FP1490A	Akoya Biosystems, Marlborough, USA	1 drop in 500 ul^a^
2°	Opal 520	HRP	Synthetic	FP1487A		1:100^a^
2°	Opal 570	HRP	Synthetic	FP1488A		1:500^a^
2°	Opal 620	HRP	Synthetic	FP1495A		1:150^a^
2°	Opal 650	HRP	Synthetic	FP1496A		1:150^a^
2°	Opal 690	HRP	Synthetic	FP1497A		1:150^a^

*Jackson IR lab* Jackson ImmunoResearch Laboratories.

^a^Antibody dilution used in automated workflow.

^m^Antibody dilution used in manual workflow.

### Determining the optimal sequence of antibodies

Sequential multiplex IHC-IF is based on consecutive labelling cycles of primary antibody, HRP polymer and TSA fluorophore. Each cycle is followed by a heat-denaturation step to remove residual antibody-HRP complexes and thereby avoid cross-reaction with HRP-conjugated secondary antibodies in subsequent staining cycles. Inadequate denaturation of the antibody-HRP conjugate may introduce background artefacts^24–26^. The use of three antibodies (α-NDUFB8, α-MTCO1 and α-pan-cytokeratin) raised in the same species (mouse) further increases the likelihood of such artefacts. Therefore, we tested effectiveness of heat-mediated denaturation by subjecting tissue sections to staining cycles for each of the four primary antibodies individually using the Opal 520 fluorophore followed by heat mediated denaturation. This was followed by a further staining cycle with the Opal 650 fluorophore, with the second primary antibody omitted. It was hypothesised that the presence of unbound residual primary antibody or inadequately denatured antibody-HRP conjugate from the first staining cycle would be revealed by the deposition of the second TSA fluorophore.

**Figure 1:**
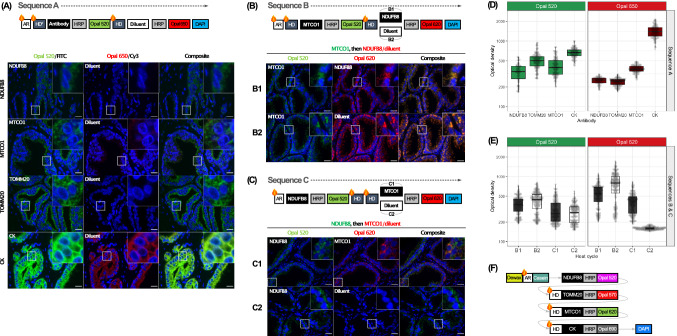
Optimisation of staining sequence. (**A**) Effectiveness of heat-mediated denaturation of each antibody-HRP complex was first individually evaluated using the staining sequence highlighted in the top panel. Using *Sequence A*, after antigen retrieval, tissue sections underwent expected heat steps for the following empirically determined staining sequence: CK > TOMM20 > MTCO1 > NDUFB8. Tissue sections were then incubated in primary antibody at optimised concentrations, then washed and incubated in species-specific HRP-conjugated polymer, followed by Opal 520 TSA substrate. To test the adequate removal of antibody-HRP complex, the sections were then incubated in antibody diluent, followed by HRP-conjugated polymer specific to the species of the subsequent staining cycle. This was followed by incubation in Opal 650 TSA substrate and DAPI. The presence of Opal 650 signal confirmed inadequate denaturation of the primary antibody-HRP complex. (**B**) The optimum sequence of the two mitochondrial antibodies raised in mouse, NDUFB8 and MTCO1, was determined by evaluating two staining sequences. In *Sequence B*, the MTCO1 staining cycle was followed by a single heat denaturation step and the NDUFB8 cycle. To test the effectiveness of heat denaturation α-MTCO1-HRP complex, the second antibody, α-NDUFB8, was replaced by incubation in the diluent. Comparable Opal 620 signal intensity was noted both with and without the inclusion of α-NDUFB8 antibody in the second staining cycle, highlighting inadequate heat denaturation of the α-MTCO1-HRP complex at all heat denaturation conditions. (**C**) In *Sequence C*, the NDUFB8 staining cycle was followed by two heat denaturation steps and the MTCO1 cycle. It was envisaged that the rabbit α-TOMM20 cycle would later be placed between the two heat denaturation steps in the multiplex staining protocol. To test effectiveness of heat denaturation of α-NDUFB8-HRP complex, the second antibody, α-MTCO1, was replaced by incubation in the diluent. Adequate heat denaturation was confirmed by the absence of Opal 620 signal using all heat denaturation conditions. Images were acquired using an Axioskop 2 epifluorescence microscope with a 40× objective and DAPI, FITC and Cy3 filters. High magnification insets (25 × 25 μm) are included. Scale bar 20 μm. (**D**) Signal intensity of Opal 520 and Opal 650 noted inadequate denaturation of CK and MTCO1 antibodies. (**E**) Signal intensity of Opal 520 and Opal 620 noted inadequate denaturation of MTCO1 demonstrated by strong Opal 620 signal in sequence B2, and adequate denaturation of NDUFB8 demonstrated by the loss of Opal 620 signal in sequence C2. Each point represents individual epithelial cells from four representative regions. (**F**) The optimised staining sequence. AR: Antigen retrieval; HD: Heat-mediated denaturation.

Profound Opal 650 fluorescence was noted in tissue sections stained with α-pan-cytokeratin antibody, with a faint fluorescent Opal 650 signal also noted in sections stained with α-MTCO1 antibody, suggesting inadequate heat-mediated denaturation of these antibody-HRP complexes (Fig. 1A,D). No detectable Opal 650 signal was observed in tissue sections stained with α-NDUFB8 and α-TOMM20 antibodies. Given the inadequate denaturation of α-pan-cytokeratin antibody-HRP complex, the pan-cytokeratin staining cycle was placed at the end of the staining sequence at position 4. Since pan-cytokeratin is used as an epithelial cell marker and not for quantification purposes, any artefactual signal from prior staining cycles probing less abundant mitochondrial targets is unlikely to influence planned downstream image analysis for cell phenotyping.

In light of the inadequate denaturation of the α-MTCO1 antibody-HRP conjugates using standard heat-mediated denaturation conditions and the need for accurate quantification of both α-MTCO1 and α-NDUFB8 targets, the sequence of NDUFB8 and MTCO1 staining cycles and various denaturation conditions was further evaluated. This included a variety of temperatures and incubation periods in CC2 reagent. When the MTCO1 cycle was placed prior to NDUFB8 (Fig. 1B,E), inadequate denaturation was observed in all conditions, further supporting the need to place the MTCO1 cycle later in the staining sequence. Adequate denaturation of α-NDUFB8 was noted when the NDUFB8 cycle was placed prior to the MTCO1 cycle (Fig. 1C,E), across all denaturation conditions (Supplementary Fig. 4).

Given robust heat-mediated denaturation of the α-NDUFB8 antibody-HRP conjugate in Sequence A under standard conditions (CC2 95 °C for 8 min), and the low abundance of the NDUFB8 target antigen, the NDUFB8 cycle was placed at position 1 of the staining sequence. This also helped minimise potential degradation of the target antigen during subsequent heat-mediated denaturation steps. The α-TOMM20 antibody, raised in rabbit, was placed at position 2 between NDUFB8 and MTCO1 cycles to provide an additional subsequent heat-mediated denaturation step, prior to probing with the anti-mouse OmniMap HRP conjugated polymer in the MTCO1 cycle at position 3.

Once the final optimum mIF staining sequence was determined, primary antibodies were paired with fluorophores based upon relative target abundance of each primary antibody estimated by signal intensities at single-plex IF staining with Opal 520, and relative quantum efficiency of spectrally separated fluorophores. An optimised sequencing strategy was devised with antibody cycles in the following order: NDUFB8, TOMM20, MTCO1, and finally pan-cytokeratin (Fig. 1F, Table 2). Notably, these optimisation experiments enabled the correct sequencing of primary antibodies of the same isotype (Mouse IgG1 α-NDUFB8 and α-pan cytokeratin).

**Table 2:** Final optimised sequence of multispectral immunofluorescence staining.

Cycle	Primary antibody	Amplification system	Fluorophore
1	NDUFB8 (Anti-Ms IgG1, 60 mins)	Anti-mouse HQ (16 mins), followed by anti-HQ HRP (16 mins)	Opal 520 (8 mins)
2	TOMM20 (Anti-Rb IgG, 30 mins)	OmniMap anti-rabbit HRP (16 mins)	Opal 570 (8 mins)
3	MTCO1 (Anti-Ms IgG2a, 60 mins)	OmniMap anti-mouse HRP (16 mins)	Opal 620 (8 mins)
4	Pan-CK (Anti-Ms IgG1, 30 mins)	OmniMap anti-mouse HRP (16 mins)	Opal 690 (8 mins)
5	Spectral DAPI (8 mins)	–	–

### Quality testing and reproducibility

**Figure 2:**
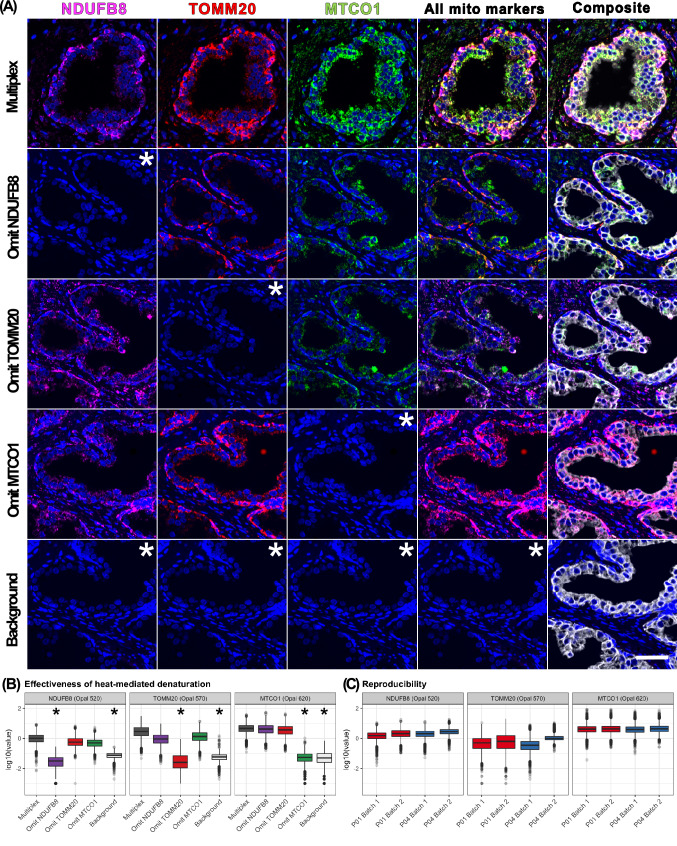
Validation of heat-mediated denaturation and reproducibility of automated multiplex OXPHOS assay. (**A**) Individual primary antibodies were sequentially omitted and replaced with incubations in diluent to evaluate effectiveness of heat-mediated denaturation of the antibody-HRP from the previous staining cycle. Residual signal from the previous staining cycle in the subsequent fluorophore channel would represent inadequate denaturation. Representative images are shown, including multiplex (all antibodies included), background (all mitochondrial antibodies omitted) and single drop-out controls. Asterisk denotes experiments with expected low fluorescence intensity. Scale bar 50 μm. (**B**) Quantitative single cell analysis of epithelial cells (n = 32,816) from 10 randomly selected regions confirmed adequate heat-mediated denaturation of NDUFB8, TOMM20 and MTCO1 antibody-HRP complexes. Asterisk denotes experiments with expected low fluorescence intensity. (**C**) Consistent signal intensity of mitochondrial markers noted across adjacent tissue sections from two patient samples (P01, P04) stained in two separate batches, confirming good reproducibility of the assay.

Following optimisation of the antibody sequence the effectiveness of heat-mediated denaturation was once again tested in a full multiplex setting by sequentially omitting individual primary antibodies and assessing any residual signal from the previous antibody cycle in comparison with background signal (Fig. 2A,B). The absence of residual signal from the previous antibody cycle confirms effective denaturation and validation of the final multiplex panel.

Both visual assessment of multiplex images (Fig. 2A) and quantitative evaluation of single cell data (Fig. 2B) confirmed minimal signal upon omission of the associated antibody, which was comparable with the background signal where all mitochondrial antibodies had been omitted. For example, in Fig. 2B, upon omission of the α-TOMM20 antibody (second staining cycle), any residual α-NDUFB8 antibody-HRP complex from the first staining cycle would catalyse the deposition of Opal 570 TSA substrate. However, Opal 570 signal intensity in this experiment was comparable with the background signal intensity, where all mitochondrial antibodies had been omitted. This represented a signal reduction by two orders of magnitude. Similarly, omission of the α-MTCO1 antibody (third staining cycle) abolished fluorophore signal in the Opal 620 channel to background levels, suggesting absence of residual α-TOMM20 antibody-HRP complex from the second staining cycle after denaturation. Thus, quantitative single cell analysis confirmed effective denaturation of α-NDUFB8 and α-TOMM20 antibody-HRP complexes, supporting reliable quantification of all three mitochondrial markers using this multiplex assay.

A key advantage of automation is the potential improvement in reproducibility of results. We therefore tested assay reproducibility across staining batches by staining serial sections from two benign prostate tissue samples on separate runs using the same batch of antibodies, HRP- conjugated polymers and fluorophores. Quantitative single cell analysis noted comparable signal intensity across both batches, confirming high reproducibility of the automated approach (Fig. 2C).

### Mitochondrial alterations across benign and malignant prostate tissues

We posited that stochastic accumulation of mitochondrial DNA mutations and clonal selection forces would together lead to widespread cellular, spatial and inter-patient heterogeneity in OXPHOS protein expression in prostate tissue. Tissue sections from five patients with prostate cancer and five age-matched patients with benign prostate histopathology were subjected to the automated workflow. Given that the accumulation of OXPHOS defects are associated with advancing age in a number of benign human tissues^3,27–31^, we included tissue samples from five patients aged 45 years or younger with benign prostate histopathology to provide a relative measure of “normal” mitochondrial protein abundance.

Since each cell contains many mitochondria, and OXPHOS defects lead to an increase in mitochondrial biogenesis^32^, we normalised single-cell level OD-NDUFB8 (OD: Optical density) and OD-MTCO1 to a mitochondrial mass marker, OD-TOMM-20. This pre-processing approach helped overcome the potential bias due to varying mitochondrial mass. Z-scores were calculated from single cell optical density data, using control data from a cohort of patients aged 45 years or younger with benign prostate histopathology.

**Figure 3:**
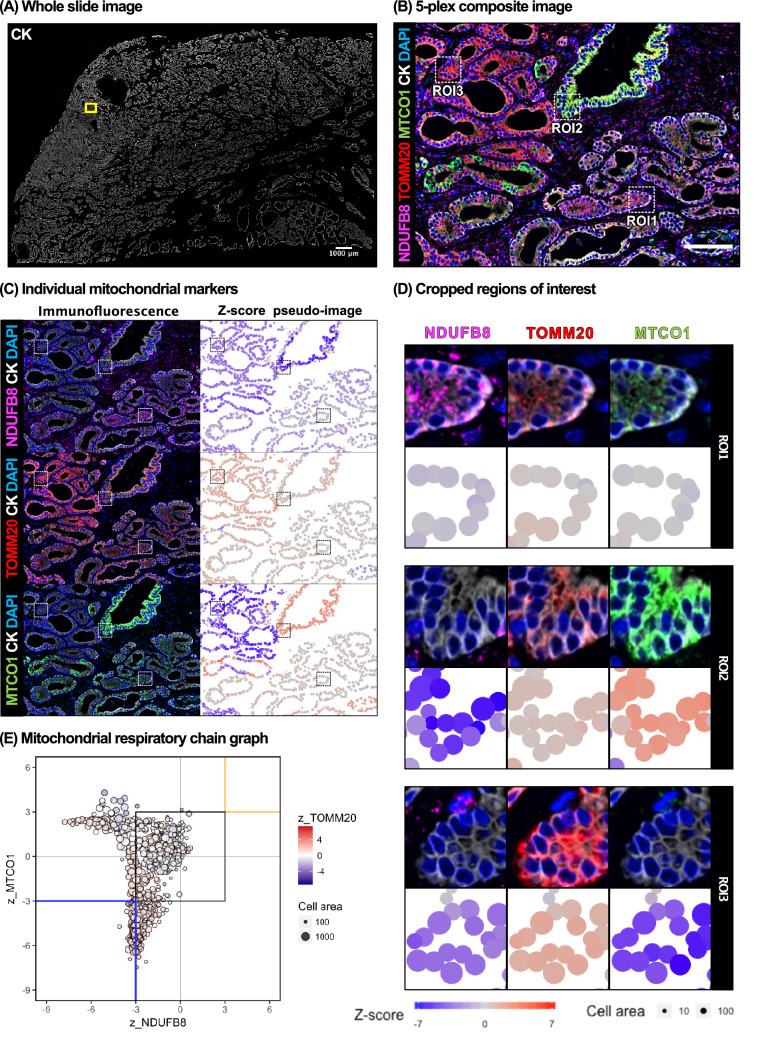
Cellular heterogeneity in OXPHOS protein abundance in prostate cancer tissue. (**A**) A region of interest was selected from an overview pan-cytokeratin map of a prostate cancer tissue section (patient P13) labelled using the final optimised multispectral staining protocol. (**B**) Composite image generated from multispectral imaging and spectral deconvolution noted spatial variations in raw NDUFB8 and MTCO1 abundance. Single cell level OD-NDUFB8 and OD-MTCO1 were obtained following automated tissue and cell segmentation. Scale bar represents 100 μm. (**C**) Immunofluorescence and pseudo-colour Z-score images of each mitochondrial marker demonstrates spatial heterogeneity in protein abundance. (**D**) Cropped regions of interest from panel (**B**) represent three cell phenotypes: normal mitochondrial protein abundance (ROI1), isolated NDUFB8 defects (ROI2), and combined NDUFB8 and MTCO1 defects (ROI3). (**E**) Mitochondrial respiratory chain (MRC) graph. Single cell level OD-NDUFB8 and OD-MTCO1 data were log-transformed and normalised for variable mitochondrial mass using OD-TOMM20. Z-scores were calculated for each of the mitochondrial markers, using data from benign prostate tissue from patients aged ≤ 45 years as controls. Each spot represents an individual epithelial cell (n = 1320 cells) from the region of interest in panel (**B**). The size of each spot correlates with cell area (in pixels). MRC graphs stratified by individual patients are presented in Supplementary Fig. 5.

Cellular heterogeneity in mitochondrial protein abundance was visualised by constructing Z-score pseudo-images. Figure 3A,B focus on one tumour specimen from patient P13 and one region within this specimen labelled with the mitochondrial multiplex panel. Figure 3C,D highlight significant spatial heterogeneity within this region and pseudo-images highlight cells with high (red) or low (blue) abundance of NDUFB8, MTCO1 or TOMM20. Cellular heterogeneity appeared to emerge in a clonal manner across representative regions of interest (ROI) highlighted in Fig. 3E. Though the vast majority of cells demonstrated ‘normal’ OXPHOS protein abundance (e.g. ROI1), we also observed cells with either low NDUFB8 abundance (e.g. ROI2) or low MTCO1 abundance and, rarely, a reduction of both NDUFB8 and MTCO1 abundance occurring with an associated increased TOMM20 abundance (ROI3). These OXPHOS phenotypes are highlighted further in Fig. 3E where single cell analysis was performed and Z-scores of NDUFB8 and MTCO1 were plotted against each other showing the cellular variation in OXPHOS phenotypes found within this sample.

**Figure 4:**
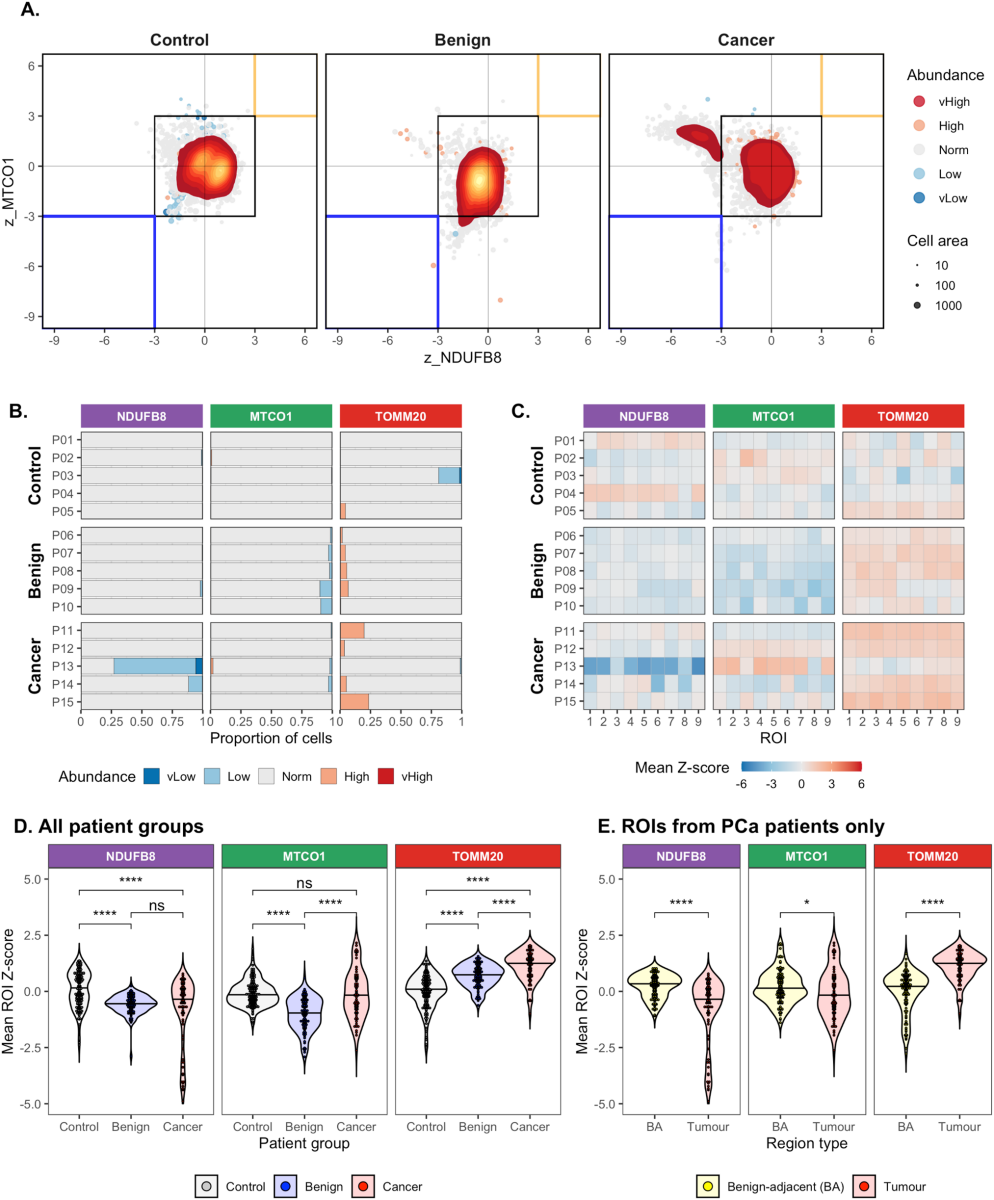
OXPHOS defects in benign and malignant human prostate tissue. Cells from each patient were classified based on mitochondrial protein abundance (median 14,670 cells per patient, 216,731 cells in total). (**A**) Mitochondrial respiratory chain (MRC) graphs depicting 500 random samples per patient and density plot including all epithelial cells across all patients within each patient group. Tissue samples from patients with no histological evidence of prostate cancer, under 45 years of age, were used as controls. Patients with benign disease were age-matched to patients with tumour tissue. (**B**) Proportion of cells in each abundance category stratified by individual patients. (**C**) Mean Z-score across all epithelial cells within 9 representative ROIs from each patient group. Mean single cell expression of NDUFB8, MTCO1 and TOMM20 across spatially disparate ranges of benign, cancerous and control prostate tissue (n = 5 patients in each group). Widespread heterogeneity both within and between patient cohorts was noted, with MTCO1-deficient cells were observed more frequently in the benign cohort, whereas NDUFB8-deficient cells were observed most frequently in a subset of patients from the cancer cohort. (**D**) Variation in mean Z-scores across each patient group (n = 309 ROIs from 15 patients). Note that only tumour regions from PCa patients were selected for this comparison. (**E**) Marker expression across tumour and benign–adjacent (BA) regions from prostate cancer patients (n = 204 ROIs from 5 patients). In panels (**D**) and (**E**), each point represents mean Z-score for all epithelial cells contained within an individual region of interest. Kruskal–Wallis test: NS, not significant; *p < 0.05; ****p < 0.0001.

Mitochondrial respiratory chain graphs were generated by plotting Z-scores for NDUFB8 expression against MTCO1 expression, and cells with Z-score < − 3 were deemed to be deficient in the relevant marker (Fig. 4A). Widespread heterogeneity both within and between patient cohorts was also noted. Frequency of MTCO1-deficient cells was greatest in older benign prostates (4.52%), compared with prostate tissue from young men (0.277%, △4.25%, *p* < 0.001), and age-matched PCa tissue samples (1.38%, △3.14%, *p* < 0.001). In contrast, NDUFB8-deficient cells were observed more frequently in PCa tissue samples (15.68%), compared with prostate samples from young men (0.23%, △15.44%, *p* < 0.001) and age-matched prostate samples (0.61%, △15.07%, *p* < 0.001). Further interrogation of these NDUFB8 deficient cells found them to originate from 2 out of the 5 PCa patients, P13 and P14, (Fig. 4B), where 72.86% and 11.27% cells were NDUFB8 deficient, respectively. A heatmap of mean Z-scores from representative regions of interest noted widespread NDUFB8 deficiency across all regions from P13, and only two regions from P14 (Fig. 4C).

Conversely, cells with high TOMM20 gradually increased from young controls (0.82%) to older benign (4.26%) and aged matched malignant prostate cells (11.87%) (Fig. 4D). Widespread cellular heterogeneity was also noted in OXPHOS protein abundance across spatially disparate regions of benign and malignant tissue regions within the cancer patient specimens particularly with NDUFB8 being lower and TOMM20 being higher in malignant cells compared to benign adjacent cells (Fig. 4E). In comparison to benign–adjacent regions, malignant regions had significantly greater proportion of NDUFB8-deficient cells (15.68% vs. 0.059%, △15.62%, *p* < 0.001), a greater proportion of MTCO1-deficient cells (1.38% vs. 0.16%, △1.22%, *p* < 0.001), and greater proportion of cells with high TOMM20 (11.87% vs. 6.49%, △5.38%, *p* < 0.001). Alterations in the three mitochondrial markers predominantly occurred in isolation, with multiple alterations occurring in rare subsets (Supplementary Figs. 5–6). Together, these findings suggest downregulation of mitochondrial complexes I & IV and increased mitochondrial mass in malignant regions compared to benign–adjacent regions in PCa tissues.

### Widespread intra-patient spatial heterogeneity in mitochondrial protein abundance

**Figure 5:**
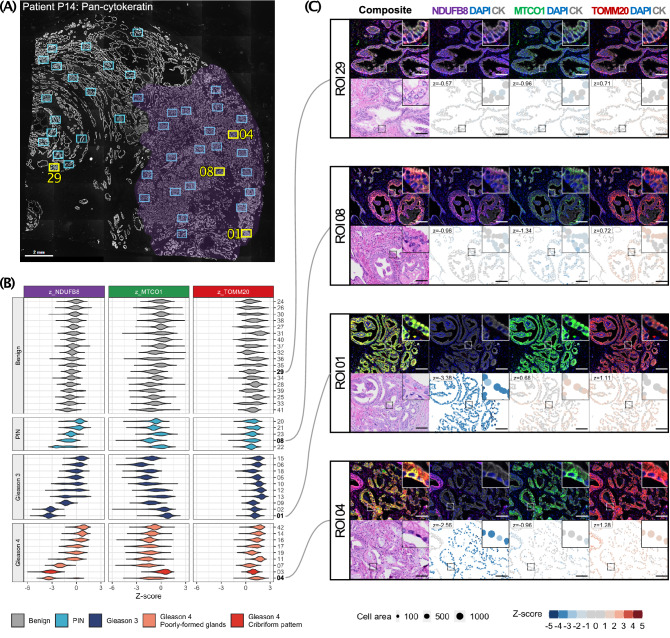
Intra-patient heterogeneity in mitochondrial marker expression. Characterisation of randomly selected regions of interest (ROIs) from patient P14. (**A**) ROIs annotated on a pan-cytokeratin labelled immunofluorescence image (4× objective, scale bar represents 2 mm). The purple region highlights tumour-rich tissue. Each blue/yellow box represents a ROI with adjacent numbers labelled for ROIs selected in panel (**C**). (**B**) Single-cell level quantification of mitochondrial marker abundance across all ROIs within P14 demonstrates widespread spatial variation amongst tumour ROIs, sorted by NDUFB8 Z-score and histopathological subgroup (n = 39,609 cells from 42 ROIs). (**C**) Representative ROIs from each histopathological subgroup. Each panel includes a top row of fluorescence-labelled images. The lower row contains corresponding haematoxylin & eosin stained images and pseudo-images representing single-cell level z-scores in epithelial cells, with ROI mean Z-score denoted above. High magnification insets (50 μm × 50 μm) are included. Scale bar represents 100 μm.

Previously (Fig. 4C), we noted significant heterogeneity in mitochondrial protein abundance in two prostate cancer specimens (patient P13 and P14). Herein we studied patient P14 in more depth by imaging mIF ROI across the tumour area highlighted by boxes in Fig. 5A. These regions were then reviewed by a histopathologist using the same tissue section but re-stained for H&E. These regions were categorised based upon histopathological features: benign–adjacent glands, prostate intra-epithelial neoplasia (PIN), Gleason 3 pattern, and Gleason 4 pattern. Single cell analysis for all these regions was performed and OXPHOS protein abundance is shown in Fig. 5B with representative regions of interest highlighted in Fig. 5C. As noted previously, there is considerable heterogeneity within the tumour itself and benign–adjacent regions within the same specimen. Areas of low NDUFB8 and high TOMM20 were observed within areas with Gleason pattern 3 and 4 PCa, compared to benign–adjacent and PIN regions. Validation of these findings in larger patient cohorts is warranted.

## Discussion

In this study we describe the development of a multiplex immunofluorescence pipeline for the evaluation of mitochondrial alterations in formalin-fixed prostate tissue specimens at the single cell level. Using drop-out controls, we validated heat-mediated denaturation of antibody-fluorophore conjugates at each staining cycle, thereby improving the accuracy of estimating the abundance of each epitope.

### Benefit over manual multiplex IF methods

Standard multiplex immunofluorescent staining protocols are often based on primary antibodies directly conjugated fluorophores or fluorophore conjugated secondary antibodies such as AlexaFluor fluorophores which require the selection of a combination of antibodies, with careful consideration of their host species and isotypes of the primary antibody. These issues limit both the sensitivity, due to the lack of signal amplification, and specificity, with the effect of limiting the number of targets that can be probed in a multiplex assay. In contrast, TSA-based protocols employ sequential staining methods, whereby each cycle of primary antibody, HRP-linker and TSA fluorophores are followed by a heat-mediated deactivation of the antibody complex. This provides opportunity for greater multiplexing, though introduces the risk of an inadequately inactivated residual complex of primary antibody and HRP from a previous staining cycle binding to the subsequent fluorophore, thereby contaminating the signal. To mitigate these risks, as supported by previous similar studies^24,26^, we recommend a series of optimisation experiments to determine the optimum sequence of antibody cycles. Here we used the following rationale: (1) NDUFB8 was placed in the first sequence due to the low abundance of the target protein to minimise any potential degradation of target antigen during subsequent heat-mediated denaturation steps, (2) the polyclonal anti-rabbit TOMM20 antibody staining sequence was placed between NDUFB8 and MTCO1 cycles to minimise risk of cross-reactivity between the two anti-mouse antibodies, and (3) MTCO1 and pan-cytokeratin were placed at the end of the sequence due to inadequate heat-denaturation of the MTCO1 and pan-cytokeratin antibodies, without any substantial impact on tissue classification and cell segmentation accuracy (Supplementary Fig. 7). Our study therefore highlights a need for careful and thorough assay optimisation.

A further potential hurdle in multiplexing fluorophores is the risk of signal crossover in multiple detection channels due to spectral overlap between fluorophores. The following approaches may help minimise this problem: (1) use of narrow-range detection filters which may consequently also lower the signal to noise ratio, (2) limit markers in each multiplex to the number of detection filters, with the caveat that larger panels will require multiple tissue sections limiting the ability to assess co-expression of multiple target proteins, (3) use fluorophores with narrow emission range (eg quantum dots), which currently provide low long-term stability, (4) use a laser light source with narrow excitation wavelengths, or (5) perform spectral imaging and linear unmixing to estimate the contribution of overlapping fluorophores to the cumulative signal intensity at each pixel. In our pipeline we used the latter approach using the Vectra 3 quantitative pathology multispectral imaging platform coupled with spectral unmixing using the inForm tissue analysis software (Akoya Biosciences) for quantitative single cell analysis.

### Automated workflows for immunophenotyping

The automated staining approach resulted in comparable results as the conventional manual approach (Supplementary Fig. 8), however reduced hands-on time for manual staining from two days down to a fully automated 14-h protocol, including a conservative 1-h set-up period, which can feasibly be run overnight. Once stained, tissue sections were scanned using the robotic slide loader coupled with the Vectra 3 system, which can be programmed to process up to 200 slides in an automated manner with minimal human input. The automated staining and imaging platforms employed in our workflow allow improved reproducibility compared to manual methods. We anticipate the reproducibility may be further improved using pre-diluted antibodies and fluorophores, in addition to the use of strict quality control measures such as standardised tissue section thickness and fixation protocols. Similarly, the use of an automated mIF pipeline has recently been reported by Pulsawatdi et al.^25^, using the Leica Bond autostainer coupled with the Vectra Polaris multispectral imaging platform. Here we demonstrate that mIF pipelines can be adapted to multiple different platforms, with the use of a Ventana Discovery Ultra autostainer and a Vectra 3 imaging platform, highlighting the potential to use automated platforms that are already in-house to develop and benefit from automated multiplex workflows.

Several alternative multiplex imaging modalities are available, including CyTOF and CODEX. Our group have recently validated the use of CyTOF for studying mitochondrial alterations across complexes I-V in muscle^6^ and brain tissues^7^. Imaging CyTOF offers the opportunity to study over 37 markers with high signal-to-noise ratio in a small representative region of tissue at 1 μm spatial resolution using heavy metal conjugated antibodies. Image acquisition by ultraviolet laser ablation of 1 mm^2^ area takes about 80 min, limiting the ability to adequately capture intra-patient heterogeneity across whole slide images without incurring high equipment running time costs. In contrast, the CODEX (CO-Detection by indexing) platform is a spatially resolved, multiplex immunofluorescence solution enabling the capture of 40 + proteins at single cell resolution in a tissue sample using antibodies tagged with a specific fluorophore conjugated oligonucleotide barcode reporters, in multiple sequential three-plex reveal-image-remove cycles. The benefit of this platform is the detection of a large number of proteins and improved spatial resolution of 250 nm (reviewed in Tan et al. 2020^33^). However, similar to CyTOF, CODEX has a limited imageable area dependent on microscope-specific CODEX stage, with a maximum area of 15.2 × 15.2 mm^2^ in 3.5 h, again limiting the ability to detect intra-patient heterogeneity. Throughput of both CyTOF and CODEX is limited by high cost of imaging and the unavailability of an automated slide loader comparable to the 200-slide loader available with the Vectra 3 platform. A key advantage over both CODEX and CyTOF is that mIF enables the user to assay much wider tissue areas to better capture intra-patient heterogeneity, and also allows the study of numerous samples at a more affordable cost and within an acceptable timescale. Nevertheless, mIF is limited to 8 markers with increasing number of markers increasing the risk of signal spill over and reducing accuracy of estimating protein abundance. Therefore, at present, the cost effectiveness and practicality of these new highly multiplex approaches for detailed multi-marker spatial analysis is unclear. These highly multiplex approaches may therefore be better suited for discovery research and allow deeper phenotyping at the single cell level. In comparison, the mIF approach presented here makes use of platforms already in use within a clinical pathology workflow. The protocols outlined in this manuscript offers an alternative practical approach for pathological multi-marker reporting at high throughput.

### Evaluating OXPHOS alterations in human PCa

Applying this methodology to evaluate human prostate tissue specimens, we noted increased frequency of complex I deficient cells and increased mitochondrial mass in malignant tissues. This is consistent with previous proteomic^8^, metabolic^34^ and genomic studies^35^, which also noted comparable Complex I defects. A compensatory increase in mitochondrial mass was also noted, as previously reported in PCa tissues^36^. Similarly, aged benign prostate tissue was found to have increased frequency of Complex IV alterations as compared to young control prostate tissue, consistent with age-related accumulation of cells deficient in Cytochrome *c* oxidase noted in prostate^37^ and other tissues^1,31,38^. However, previous proteomic studies have largely lacked single cell level spatially-resolved data and been unable to adjust for altered mitochondrial mass in the estimation of mitochondrial protein complex abundance. Therefore, the true burden of OXPHOS defects in PCa may have been underestimated in prior studies. We address this potential bias by adjusting Complex I and IV protein abundance for mitochondrial mass using the TOMM20 marker.

We observed remarkable spatial heterogeneity in oxidative metabolism in PCa tissues, as previously observed at a transcriptomic level^39,40^. Though some of this variation may be due to variation in Gleason grade, areas with comparable histopathology also tended to have variable OXPHOS protein abundance raising the possibility that metabolic alterations may occur without obvious morphological change. This finding may help sub-stratify patients with similar histopathological grade but diverse long-term outcomes, based on OXPHOS protein abundance. However, the small patient cohort used in this pilot study was underpowered to test this hypothesis. Since pan-cytokeratin was used as a marker for all epithelial cells, we were unable to deep phenotype tumour cell subsets to study whether OXPHOS protein abundance varied between basal and luminal cell types. Nevertheless, as the cell type marker is placed in the final staining cycle, the protocol can be easily amended for use of other cell type specific markers.

In conclusion, we described the development and optimisation of a robust automated mIF protocol to assess mitochondrial defects in archival formalin-fixed prostate tissue. Applying this approach to a small patient cohort, we demonstrate both age-related and disease-related mitochondrial alterations, occurring in a spatially heterogenous manner in malignant tissues. The use of this approach in larger archival tissue cohorts will aid greater understanding of the association between mitochondrial alterations with disease features and long-term outcomes.

## Methods

### Human prostate samples

The study was conducted according to the Good Clinical Practice guidelines and the Declaration of Helsinki. Prostate tissue samples were acquired from the Manchester Cancer Research Centre Biobank following ethical approval from the North West—Greater Manchester South Research Ethics Committee (REC#07/H1003/161+5) with informed consent from patients undergoing either radical prostatectomy (prostate cancer samples) or radical cystoprostatectomy (patients with bladder cancer but no histopathological evidence of prostate cancer). Samples were formalin fixed and embedded into paraffin blocks. All samples used in this study are described in Table S1.

### Antibodies

Antibodies and dilutions used in this study are outlined in Table 1.

Previously, these antibodies against NADH:Ubiquinone Oxidoreductase Subunit B8 (NDUFB8) and mitochondrial cytochrome c oxidase subunit 1 (MTCO1) were used as markers of Complex I and Complex IV respectively and have been validated in a cohort of patients with well characterised genetically-confirmed mitochondrial disorders^4^. They are currently routinely used in diagnostic clinical evaluation of muscle biopsy samples from patients with suspected mitochondrial diseases. We used translocase of outer mitochondrial membrane 20 (TOMM20) as a well-established marker of mitochondrial mass^23^, to normalise NDUFB8 and MTCO1 abundance for mitochondrial mass.

⍺-pan-cytokeratin antibody was used to target cytokeratins 4, 5, 6, 8, 10, 13, and 18 in prostate tissue for identification of a broad spectrum of epithelial cells, including both luminal and basal cell types.

### Manual multiplex immunofluorescence

We replicated the manual staining protocol employed by Rocha et al. 2015 with a few modifications for use with formalin-fixed prostate tissue. Briefly, paraffin-embedded tissue sections were dewaxed in Histo-Clear (National Diagnostics, USA) followed by graded ethanol solutions. Slides were placed in an antigen retrieval unit with pH 8.0 EDTA, and tissue sections were subsequently incubated in 10% normal goat serum for one hour, following which endogenous biotin was blocked (Vectastain ABC kit, 2BScientific Ltd, Oxfordshire, UK). Tissue sections were incubated in a cocktail of primary antibodies (Table 1), which included ⍺-NDUFB8, ⍺-MTCO1, and ⍺-TOMM20 at 4 °C for 16 h and then incubated in a cocktail of secondary antibodies conjugated to AlexaFluor fluorophores and biotinylated IgG1 antibody at 4 °C for 2 h. Slides were incubated in AlexaFluor 647 streptavidin-conjugated secondary antibody at 4 °C for 2 h. Subsequently, tissue sections were then incubated in ⍺-pan-cytokeratin antibody, conjugated to AlexaFluor 750, and incubated in DAPI for 15 min. Cover slips were mounted on to slides using Prolong Gold (Invitrogen, Paisley, UK). Finally, slides were visualised at 20× magnification using a Zeiss Axioimager M1 epifluorescence microscope and viewed with Zen 3 imaging software. Image processing was performed using Fiji software to obtain optical density values for each marker^41^.

### Chromogenic immunohistochemistry (IHC)

DAB (3,30-diaminobenzidine) single-plex IHC detection was carried out initially to determine the appropriate primary antibody conditions using the Ventana Discovery Ultra automated IHC/ISH research platform (Roche Diagnostics Limited, Burgess Hill, UK) before translating to the TSA immunofluorescence.

Freshly cut 4 μm tissue sections were mounted on glass slides. Sections were placed in the Ventana Discovery Ultra and dewaxed; heat induced epitope retrieval was performed using CC1 reagent for 64 min. Primary antibodies were added by manual application and the final concentrations determined are provided in Table 1. Secondary HRP complexes and DAB were completed in a fully automated manner. Slides were washed, dehydrated through a series of alcohols and coverslipped. Slides were imaged using an Aperio CS2 whole slide scanner.

### Multiplex immunofluorescence

Following initial validation of primary antibodies using DAB, the panel was further optimised with the benefits of tyramide signal amplification (TSA) with multispectral imaging using Vectra 3 system as part of a 5-plex assay. Supplementary Figure 1 outlines the final determined automated workflow used in this study.

Automated multiplex staining was performed on a Ventana Discovery Ultra platform (including all reagents unless otherwise specified, Roche Diagnostics Limited. Burgess Hill, UK), which was programmed to perform the following steps (1) dewaxing using EZPrep reagent, (2) heat-mediated antigen retrieval using CC1 reagent, (3) incubation in casein-based Discovery Inhibitor, followed by 5 sequential staining cycles. Each staining cycle included incubation of the primary antibody, followed by an HRP-linker and an Opal TSA fluorophore (Akoya Biosystems, Buckinghamshire, UK). Heat-mediated antibody denaturation was performed using CC2 reagent prior to each cycle of sequential staining to denature the primary antibody-HRP complex, and deposit secondary TSA fluorophore. This avoided the binding of subsequent fluorophores to any residual unbound HRP-conjugated primary antibody. Targets were probed using the following optimised sequence: NDUFB8, TOMM20, MTCO1, pan-cytokeratin, and then DAPI. Details are provided in Table 2.

Spectral library slides were prepared by processing tissue sections using the above multiplex protocol but omitting all but one combination of primary antibody and fluorophore. Similarly, drop out controls were generated to test effectiveness of heat-mediated antibody denaturation, whereby a single primary antibody was omitted in turn, to assess for non-specific binding of the next antibody to any residual HRP-conjugated antibody from the previous staining cycle. An autofluorescence slide was generated by using the multiplex protocol above and omitting all primary antibodies and fluorophores. Images included in the single-plex and double-plex optimisation experiments were acquired using the Axioskop 2 epifluorescence microscope (Carl Zeiss AG, Jena, Germany) with a 40× objective and a CoolSNAP HQ CCD camera (Teledyne Photometrics, Tucson, Arizona) using the Metamorph software.

### Imaging

Whole-slide scanning of haematoxylin & eosin-stained tissues sections was performed using an Olympus VS200 MTL (Olympus Tokyo, Japan), in conjunction with an Olympus UPLXAPO20X objective lens. Whole slide brightfield images and epifluorescence images were analysed using QuPath software^42^.

The Vectra 3, 200-slide system (Akoya Biosystems, Buckinghamshire, UK), comprising of a multi-spectral imaging microscope (DAPI, FITC, Cy3, Texas Red and Cy5 filter cubes), coupled to an automated slide stage and slide loader was used for image acquisition. Overview images were taken using a 4× objective, and regions of interest were imaged using a 20× objective. Exposure times were optimised for all filter cubes using a range of multiplex stained slides. Following whole-slide epifluorescence imaging, at least 10 regions of interest were selected per sample to collect multispectral images across the 440–720 nm spectral range at 20 nm intervals.

### mIF image processing

Multispectral images were linearly unmixed, and batch processed on InForm Tissue Studio v2.3 software (Akoya Biosystems, Malborough, USA) following the manufacturers protocol. Representative images from single-stained slides and the autofluorescence slide were used to generate a spectral library. Multispectral images were spectrally unmixed. Notably, contribution of autofluorescence was removed at this stage. Each image was manually quality checked for technical and tissue artefacts.

A trainable tissue segmentation algorithm was employed to determine epithelial and stromal regions using the DAPI and cytokeratin channels. Cell segmentation was performed using DAPI counterstain for nuclear identification, and ⍺-pan-cytokeratin labelling to determine cytoplasmic area. The mean signal intensity of each of the fluorophores were measured at an individual cell level. Z-scores for mitochondrial markers were calculated with reference to a control cohort comprising of patients without histopathological evidence of prostate cancer, aged 45 years or below. Therefore, Z-scores were used as a measure of relative OXPHOS protein abundance.

### Statistical analysis

Measurement of protein abundance relative to a control cohort was performed by calculating Z-scores. Single-cell level signal intensity data were used to generate Z-scores in R 3.4.3, as previously described by Rocha et at (2015)^4^. Briefly, log-10 transformation of signal intensity was first performed to achieve normality, which was confirmed by visual inspection. Data from young patients (under 45 years of age) were used as controls. Linear regression of log10-MTCO1 and log10-NDUFB8 (dependent variables) against log10-TOMM20 (independent variable) were performed using data from control patient tissue. Parameters describing the distribution of log10-TOMM20 (mean and standard deviation, SD), as well as parameters describing the linear relationship between log10-NDUFB8 versus log10-TOMM20 and log10-MTCO1 versus log10-TOMM20 were calculated. The deviation of log10-MTCO1 and log10-NDUFB8 of each cell from the expected values was used to calculate Z-scores. Z-scores for NDUFB8 and MTCO1 were categorised as follows: “very low” (Z-score <  − 6SD), “low” (Z-score <  − 3SD), “normal” (Z-score between − 3SD to + 3SD), and “high” (Z-score >  + 3SD). Z-scores for TOMM20 were categorised as follows: “low” (Z-score <  − 2SD), “normal” (Z-score − 2SD to + 2SD), and “high” (Z-score >  + 2SD). Proportions were compared between groups using the Chi square test.

## Supplementary Information

Supplementary Information.
