# Supplementary material for: Automated quantitative high-throughput multiplex immunofluorescence pipeline to evaluate OXPHOS defects in formalin-fixed human prostate tissue
Source: Sci Rep. 2022 Apr 22;12:6660. doi: 10.1038/s41598-022-10588-z (PMC9033818; doi:10.1038/s41598-022-10588-z)
Supplement: Supplementary file 1 — Supplementary Information. [file 41598_2022_10588_MOESM1_ESM.docx]

# Supplementary Information

## Supplementary tables

**Table S1 - Whole prostate blocks used in exemplar experiment.**

BCR: biochemical recurrence. R0: Negative surgical margin. R1: Positive surgical margin.

| Category | Patient | Age | Procedure | Histopathology |
| --- | --- | --- | --- | --- |
| Control | P01 | 37 years | Cystoprostatectomy | Benign prostate tissue |
|  | P02 | 37 years | Cystoprostatectomy | Benign prostate tissue |
|  | P03 | 32 years | Cystoprostatectomy | Benign prostate tissue |
|  | P04 | 41 years | Cystoprostatectomy | Benign prostate tissue |
|  | P05 | 34 years | Cystoprostatectomy | Benign prostate tissue |
| Benign | P06 | 69 years | Cystoprostatectomy | Benign prostate tissue |
|  | P07 | 64 years | Cystoprostatectomy | Benign prostate tissue |
|  | P08 | 54 years | Cystoprostatectomy | Benign prostate tissue |
|  | P09 | 69 years | Cystoprostatectomy | Benign prostate tissue |
|  | P10 | 66 years | Cystoprostatectomy | Benign prostate tissue |
| Cancer | P11 | 58 years | Radical prostatectomy | GS 3+4=7 pT3a N0 R0 BCR free at 9.8 years |
|  | P12 | 62 years | Radical prostatectomy | GS 3+3=6 pT3a N0 R1 |
|  | P13 | 59 years | Radical prostatectomy | GS 3+3=6 pT2 N0 R1 BCR at 12.3 years |
|  | P14 | 65 years | Radical prostatectomy | GS 3+3=6 pT3a N0 R1 BCR free at 12.2 years |
|  | P15 | 66 years | Radical prostatectomy | GS 3+4=7 pT2 N0 R1 BCR at 5.3 years |

## Supplemental Figures

**Supplementary Figure 1: Overview of the automated workflow.** The automated workflow comprises of four stages. (**A**) Formalin-fixed tissue sections undergo automated staining on the Ventana Discovery Ultra platform followed by (**B**) automated imaging on the Vectra 3 system. (**C**) Multispectral images were unmixed, segmented and single cell data generated using InForm Tissue Finder software. (**D**) Data linkage and analysis was undertaken on RStudio.

**Supplementary Figure 2: Optimised single-plex automated IHC-DAB and IHC-fluorescence assays.** Prostate tissue sections on the Ventana Discovery Ultra platform for IHC-DAB using the Chromogenic IHC kit, and the IHC-IF using Opal 520. Representative images of optimised antibody dilutions for NDUFB8 (1:100, anti-Ms HQ system), MTCO1 (1:500, anti-Ms OmniMap) and TOMM20 (1:500, Anti-Rb OmniMap), and appropriate isotype controls are shown. IHC-DAB sections were imaged using the Aperio CS2 whole slide brightfield scanner, whereas the IHC-IF sections were imaged using the Axioskop 2 epifluorescence microscope with a 10x objective and DAPI and FITC filters at 200ms exposure. Scale bars 50μm.


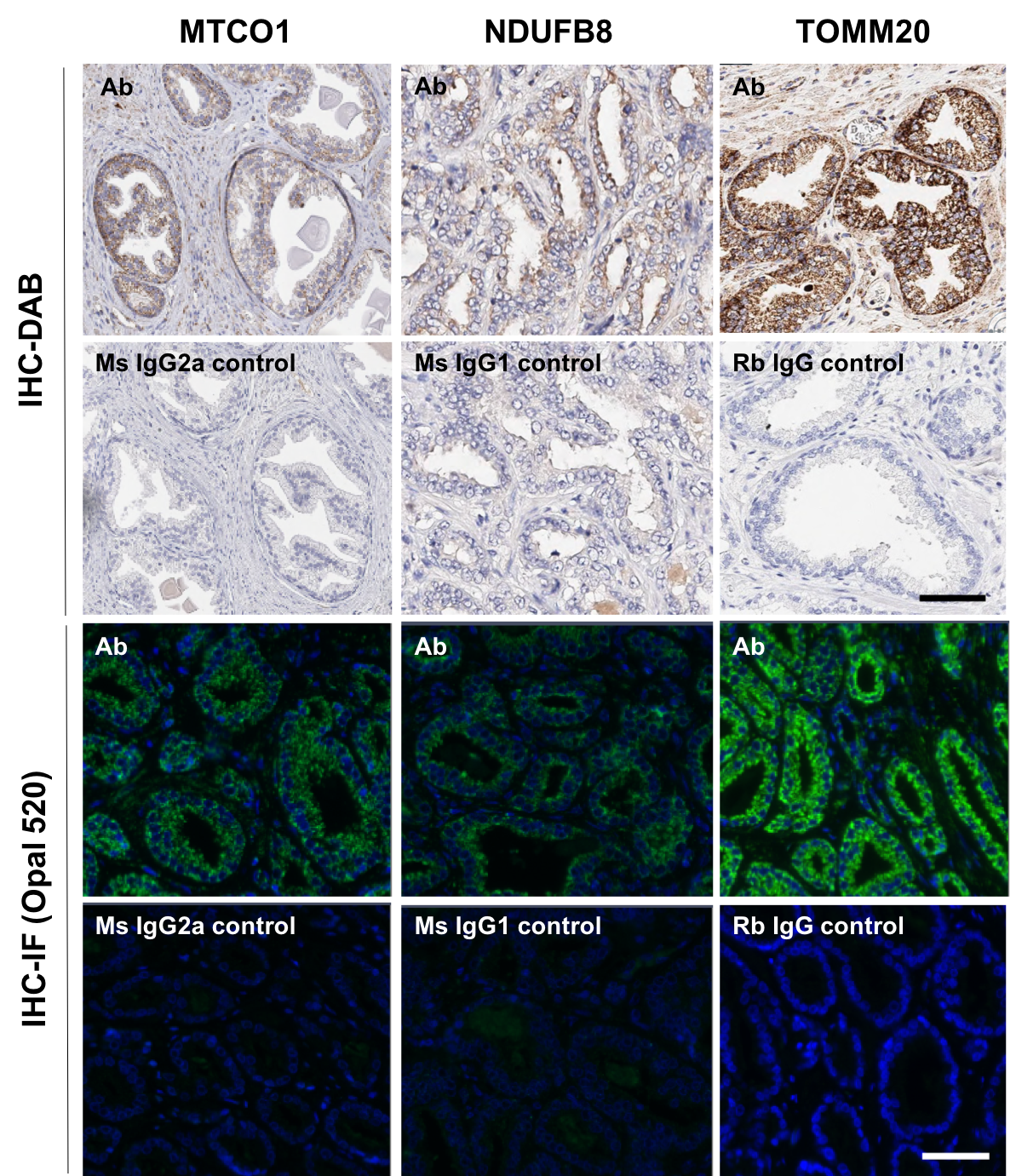


**Supplementary Figure 3**: **Optimisation of NDUFB8 labelling.** Immunofluorescence images of prostate tissue labelled with varying concentrations of α-NDUFB8 primary antibody and use of either UMap or HQ HRP for signal amplification. Opal 520 fluorophore was used at concentration 1:100 across all parameters. Scale bars 50μm.

**
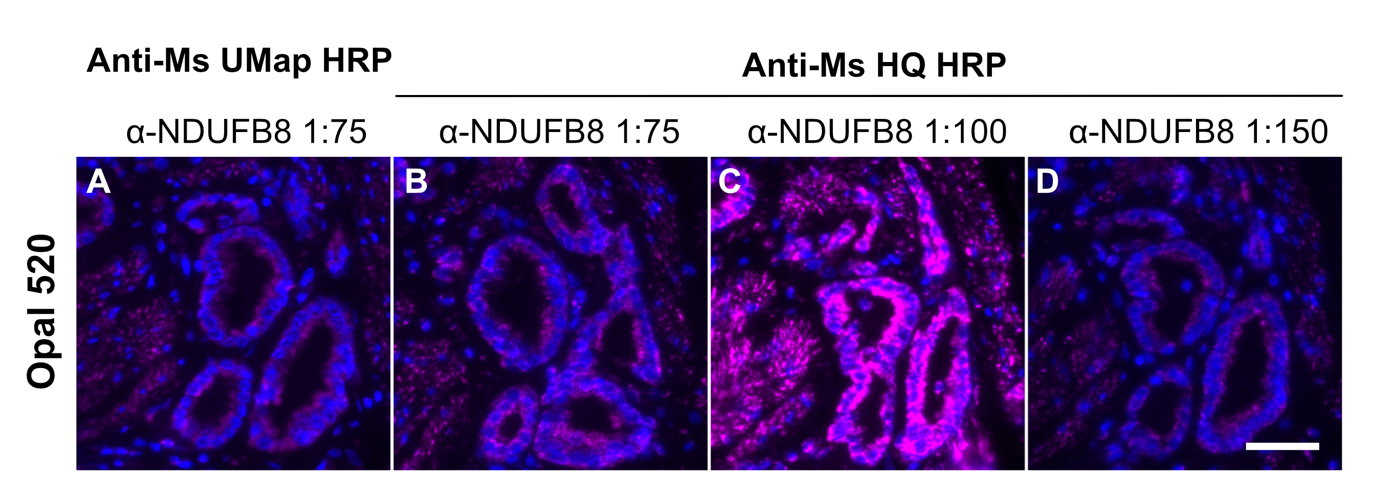
**

**Supplementary Figure 4**: **Impact of different heat denaturation conditions**. Heat-mediated denaturation of the first antibody-fluorophore conjugate was optimised across both Sequence B (MTCO1 followed by NDUFB8 cycle) and Sequence C (NDUFB8 followed by MTCO1 cycle) as described in Figures 1B and 1C, respectively. A variety of temperatures (90, 95 and 100°C) and duration of denaturation cycle (8 and 16 minutes) were tested.


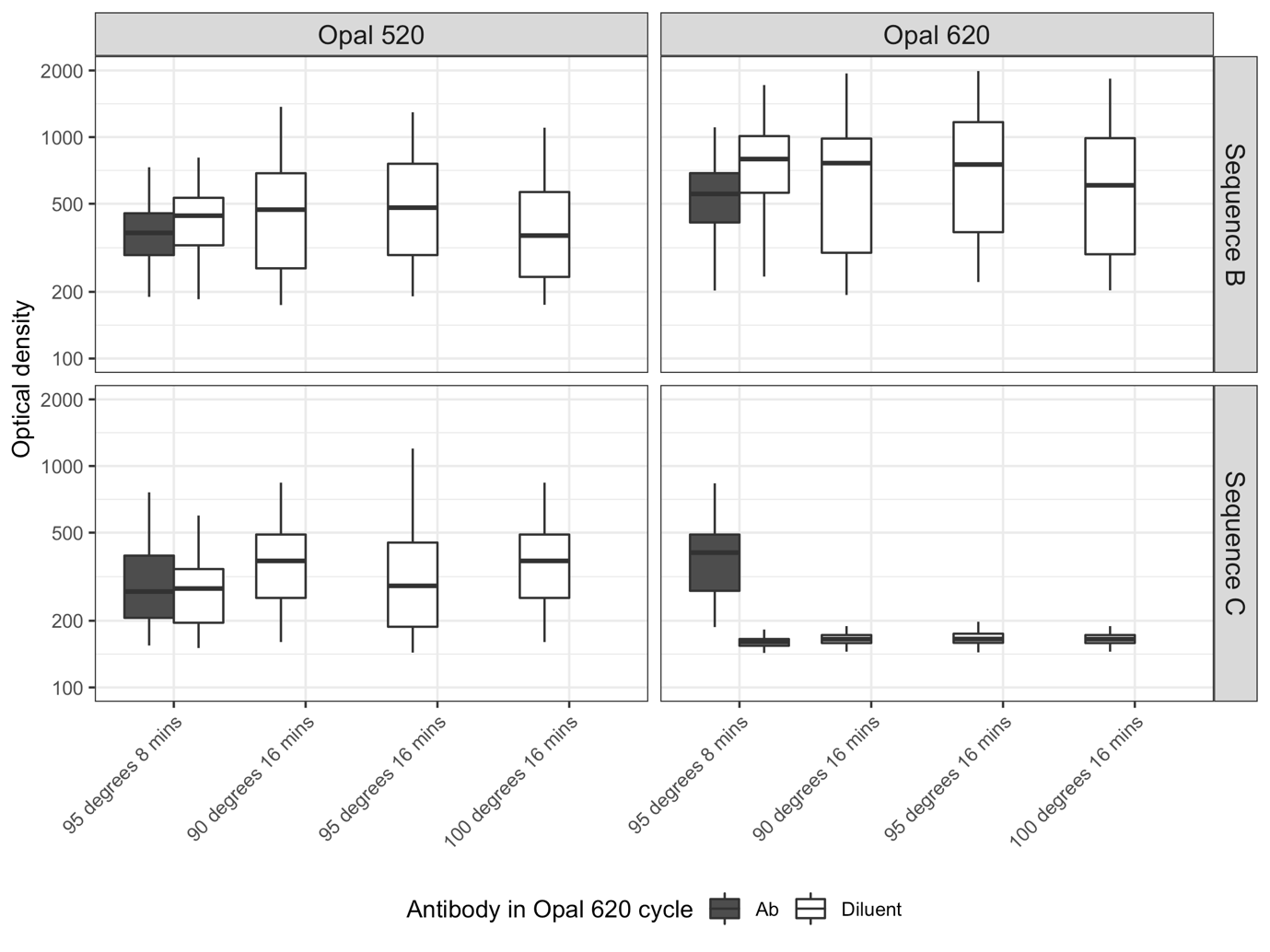


**Supplementary Figure 5: Cellular heterogeneity in OXPHOS protein abundance.** Mitochondrial respiratory chain graphs were generated from data from 15 patients with either benign prostate tissue or prostate cancer (n=5 patients in each group). Data from patients aged ≤45 years were included as a control cohort. Data from 216,731 cells are represented (median 14,670 cells per patient). Widespread intra-patient and inter-patient cellular heterogeneity in OXPHOS protein abundance was observed. For example, two distinct cell populations were noted in patients 13 and 14. Proportion of cells categorised into low, normal and high expression for each individual patient are presented in *Figure 2B*.


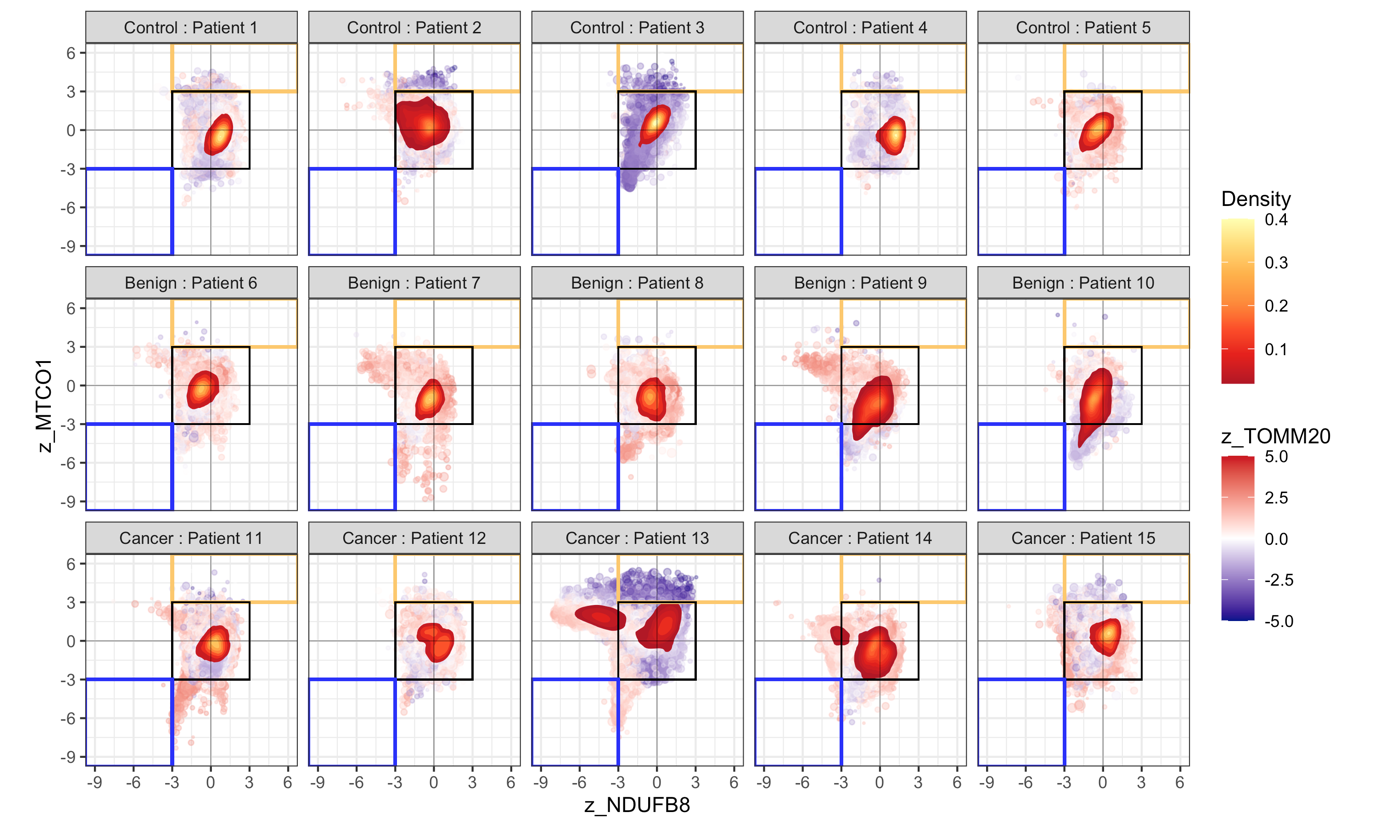


**Supplementary Figure 6: Rare incidence of cells with multiple OXPHOS alterations.** Venn diagram highlighting percentage of cells classified as either NDUBF8-low, MTCO1-low or TOMM20-high across all included (**A**) Control (n=172,163 cells), (**B**) Benign (n=102,385 cells), and (**C**) Cancer specimens (n=243,510 cells). Darker shade corresponds to higher percentage of cells within a group. Note low proportion of cells classified as both NDUFB8-low and MTCO1-low.


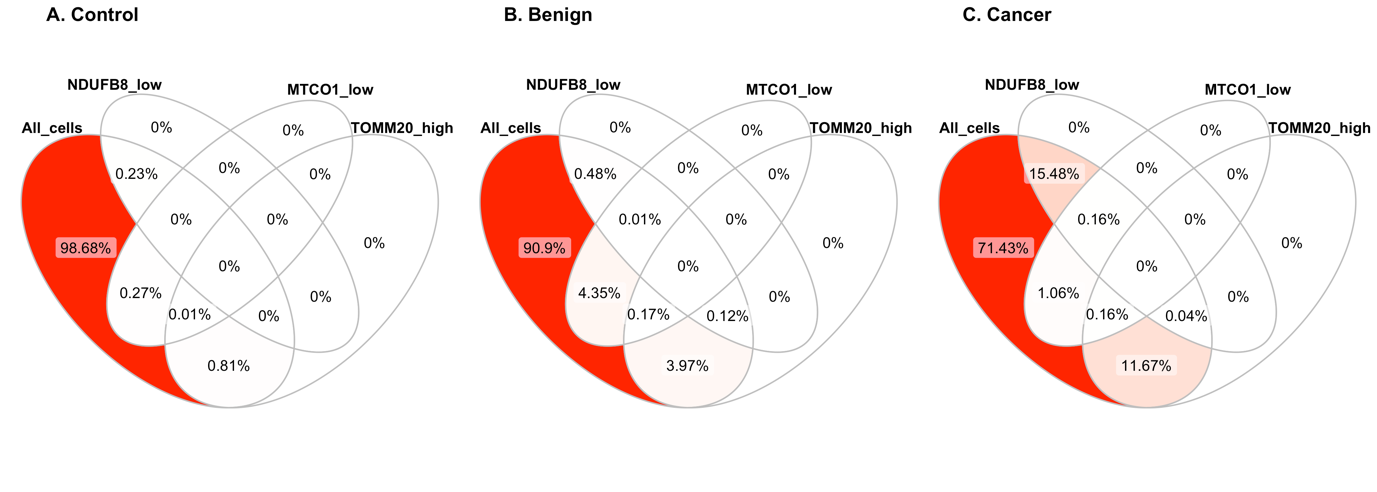


**Supplementary Figure 7: Tissue classification and cell segmentation. (A)** Representative composite multiplex immunofluorescence image from benign prostate tissue from patient P09. (**B**) Trained tissue segmentation with epithelial (red), stromal (green), and empty tissue regions (blue). (**C**) Stroma nuclear and cell segmentation, based on nuclear detection using DAPI counterstaining (green), followed by outward cell growth into surrounding cytoplasm (various colours) based on empirical cell size. (**D**) Epithelial nuclear and cell segmentation based on nuclear detection using DAPI counterstaining (green), followed by outward cell growth into surrounding cytoplasm (various colours) based on extent of pan-cytokeratin-positive epithelial regions. Scale bar 100 μm.

**
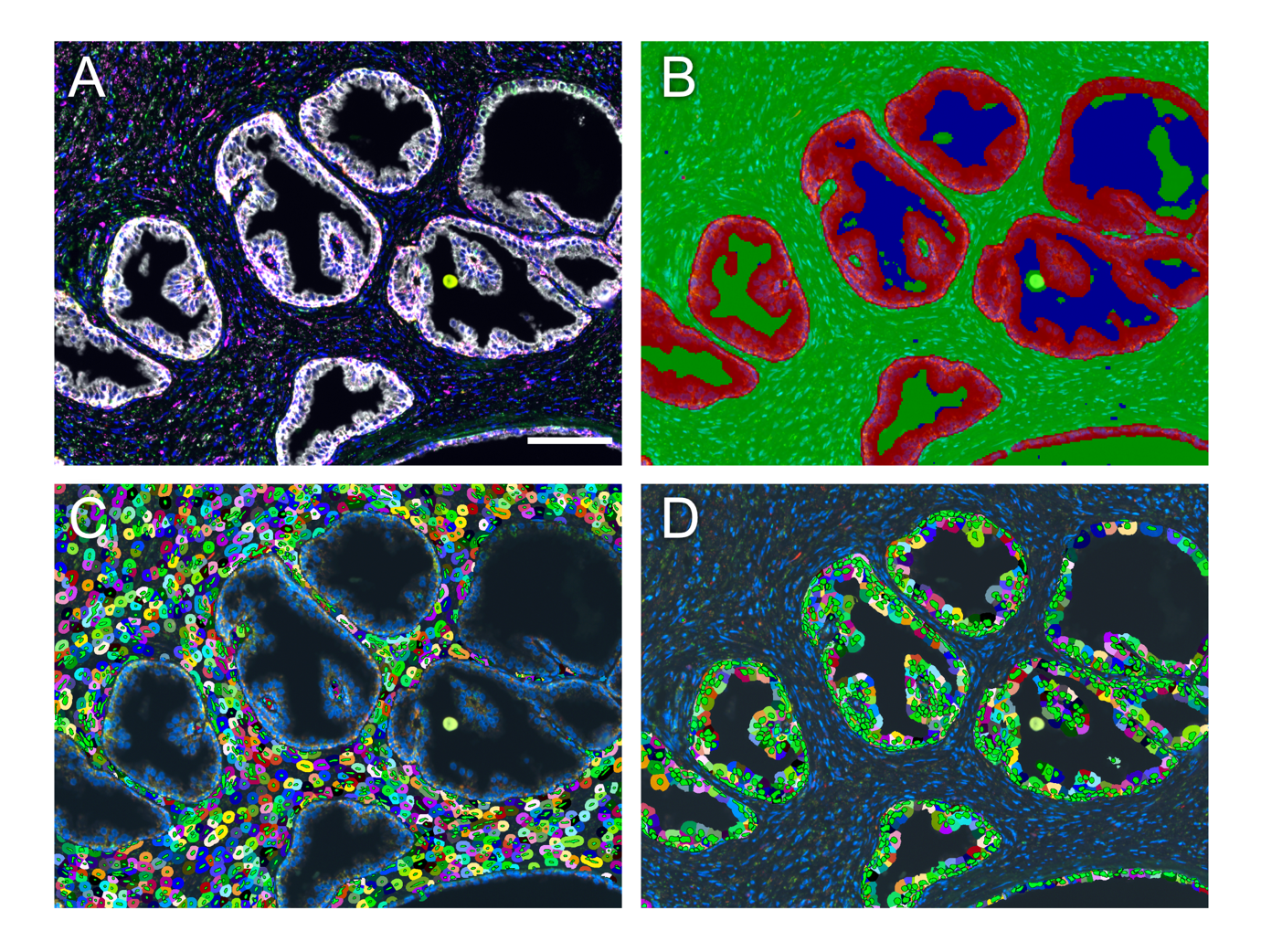
**

**Supplementary Figure 8: Comparison of automated and manual multiplex assays.** Adjacent tissue sections were subjected to automated (top row) and manual (bottom row) staining protocols. Asterix denotes a cluster of epithelial cells deficient in *NDUBF8* evident in both sections. Scale bar 50 μm.

**
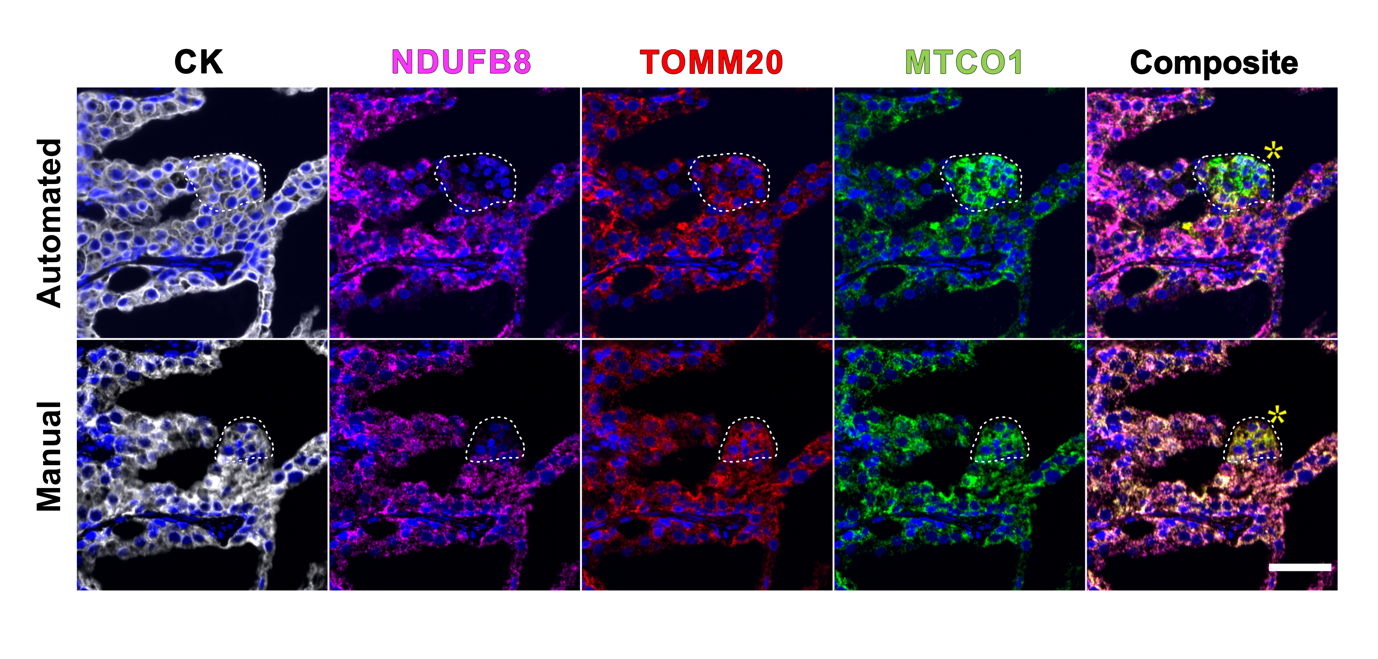
**
